# Cross-national examination of adolescent suicidal behavior: a pooled and multi-level analysis of 193,484 students from 53 LMIC countries

**DOI:** 10.1007/s00127-022-02287-x

**Published:** 2022-04-21

**Authors:** Anne Abio, Priscilla N. Owusu, Jussi P. Posti, Till Bärnighausen, Masood Ali Shaikh, Viswanathan Shankar, Michael Lowery Wilson

**Affiliations:** 1 Injury Epidemiology and Prevention (IEP) Research Group, Turku Brain Injury Centre, Turku University Hospital and University of Turku, Turku, Finland; 2 Heidelberg Institute of Global Health (HIGH), University of Heidelberg, Heidelberg, Heidelberg, Germany; 3 Department of Neurosurgery and Turku Brain Injury Center, Neurocenter, Turku University Hospital and University of Turku, Turku, Finland; 4 Department of Epidemiology and Population Health, Albert Einstein College of Medicine, Bronx, NY USA

**Keywords:** Mental health, School health, Low-income country, Middle-income country, Epidemiology, Self-harm, Adolescents

## Abstract

**Introduction:**

Suicide is a leading cause of adolescent mortality worldwide. We aimed to estimate the prevalence and identify individual-level and country-level factors which might explain the variability in suicidal behavior among students in 53 low to middle income countries.

**Methods:**

We used data on adolescents aged 12–16 years from the Global School-based Student Health Surveys from 2009–2016. The suicidal behaviors investigated included suicide ideation, suicidal planning and suicide attempt. The prevalence was estimated for 53 countries, while a multilevel logistic regression analysis (33 countries) was used to investigate the associations of these behaviors with individual and country-level contextual risk factors. The contextual variables included the Gini Coefficient, Gross Domestic Product per capita, pupil-to-teacher ratios, population density, homicide rates, law criminalizing suicide and the night light index.

**Results:**

The overall prevalence of suicide ideation, making a plan and suicide attempt were 10.4%, 10.3% and 11.0%, respectively. The highest prevalence rates reported were from the Americas. The strongest risk factors associated with suicidal behavior included anxiety, loneliness, no close friends and the substance abuse. Among the country level variables, the night light index was associated with making a suicide plan and attempting suicide.

**Conclusion:**

The non-significant country level findings were not entirely surprising given the mixed results from prior studies. Additional knowledge is thus achieved with regard to country level factors associated with suicidal behavior across adolescent populations.

## Introduction

Suicide is a leading cause of death among adolescents worldwide [47], and is the second leading mortality determinant among persons aged 15–29 [49]. While most studies on adolescent suicidal behavior have focused on high-income countries (HIC), suicide and suicidal behaviors have emerged as serious threats to adolescent health in low- and middle-come countries (LMICs) [27, 35]. The mid-point percentage prevalence of suicidal ideation among European adolescents aged 15–16 was reported at 23.5%, while being 15% among those aged 13–15 in LMICs [27]. A review found wide variations in quantifying the burden of psychological disorders among school-aged children in sub-Saharan Africa [28]. This points to a need for consistent and in-depth assessment methods to understand the prevalence of mental health problems in LMIC settings.

The onset of suicidal behavior co-occurs with adolescent development, during which the individual achieves social development and gains a fuller understanding of the finality of death, with more than one-third of adolescent ideators devising a suicide plan [33]. Over the course of adolescence as many as 63% of individuals around the world are estimated to have endorsed suicidal behavior at some point in their lives [6, 15]. It is noted that suicidal behavior heightens during the second decade of life and becomes more stable with further progression into adulthood [8, 20, 33]. There are gendered patterns in the manifestation of suicidal behavior. Although literature evidence reports that males exhibit a higher rate of lethal suicide attempts ranging from 11.1–41.1 deaths per 100,000 compared to 2.3–10.8 deaths per 100,000 by females [6, 34, 37]. Further noted is that suicidal behavior is about 1.3 times higher [16] among young, unmarried females than among males. The evidence for this observation draws from the use of more lethal methods by males such as firearms, hanging and carbon monoxide poisoning while females employ methods which may not necessarily result in death [5]. By contrast, young females in China ingest toxic agricultural agents leading to a higher rate of fatality compared to males [6]. Psychiatric autopsies from 80% of suicide cases [43] demonstrate underlying medical histories of mental disorders such as poor impulse control, personality disorders, as well as alcohol and drug dependence. Additionally, studies conducted among adolescents and young adults show that the strongest associations for suicidal risk are prior clinical diagnoses of major depressive disorder, anxiety, substance abuse, and disorderly conduct [18, 24, 42]. Suicidal behavior may be attributable to external determinants taking the form of pressures from sociocultural factors, bullying, and acute onset of stressful life circumstances[18] .

Previous studies have been conducted on suicidal behavior using the Global School-based Student Health Survey (GSHS) data in various countries [10, 22, 25, 27, 40, 46]. However, none of the studies included country level contextual factors, although GDP was considered in one study [25]. Societal or country level factors may be useful in estimating levels of social and environmental stress that individuals may face. This includes for example, income inequality, which may also ultimately influence mental health [11, 41]. Identifying contextual factors may benefit public health programs seeking to mitigate suicidal behavior. With this in mind, the present study sought to determine what country-level contextual variables may be associated with suicidal behavior in addition to individual risk factors in LMICs.

## Methods

### Study Population

We utilized GSHS data from 2009–2016. The GSHS is a cross-sectional survey carried out among adolescents in schools in LMICs. The data are collected from nationally representative samples of school-going adolescents typically aged 13–17 years. A two-stage cluster sample design was used to collect these data. Schools were selected with a probability proportional to their respective enrolment sizes. Then, classrooms were randomly selected with all students in the selected classrooms being eligible to participate. The responses to the questionnaires were self-reported by students who chose to participate. A standard questionnaire was used in all countries. A description of the survey and methodology is available elsewhere [3].

All countries that collected information on suicidal behavior were included in the analysis. Fifty-three countries were included: 7 from Africa, 15 from the Americas, 10 from the East Mediterranean, 5 from South East Asia and 16 from the West Pacific. The regions are based on World Health Organization (WHO) classifications. The school response rate ranged from 83–100% while the student response rate ranged from 60 to 99%. Ethical approval was provided by the respective governments in each country, and informed assent/consent obtained by students and/or their parents or guardians or school officials. Adolescents aged 12–16 years (*n* = 193,484) were included in the analysis. The number of students ranged from 101 in Tokelau to 28,055 in Argentina.

### Measures used

#### Dependent variables

Suicide ideation, made a suicide plan and attempted suicide were the outcome measures of interest with each having a 12-month period of recall (see Table 1). Each of the outcome variables were analyzed separately.

**Table 1:** Survey derived variables: Cross-national examination of adolescent suicidal behavior in 53 countries

Variable name	Question	Coded as
Suicide ideation	During the past 12 months, did you ever seriously consider attempting suicide?	No (0), Yes (1)
Made a suicide plan	During the past 12 months, did you make a plan about how you would attempt suicide?	No (0), Yes (1)
Suicide attempt	During the past 12 months, how many times did you actually attempt suicide?	Never (0), 1 or more times
Age	How old are you?	12–16 (continuous)
Sex	What is your sex?	Male (1), Female (2)
Hunger	During the past 30 days, how often did you go hungry because there was not enough food in your home?	Never/ Rarely/ Sometimes (0), Most of the time/Always (1)
Attacked	During the past 12 months, how many times did you get physically attacked?	Never (0), 1 or more times (1)
Physical fight	During the past 12 months, how many times were you in a physical fight?	Never (0),1 or more times (1)
Injured	During the past 12 months, how many times were you seriously injured?	Never (0), 1 or more times (1)
Bullying victimisation	During the past 30 days, on how many days were you bullied?	Never (0), 1 or more times (1)
Loneliness	During the past 12 months, how often have you felt lonely?	Never/ Rarely/ Sometimes (0) Most of the time/Always (1)
Anxiety	During the past 12 months, how often have you been so worried about something that you could not sleep at night?	Never/ Rarely/ Sometimes (0), Most of the time/Always (1)
Truancy	During the past 30 days, on how many days did you miss classes or school without permission?	0 to 2 days (0), 3 or more days (1)
Helpful peers	During the past 30 days, how often were most of the students in your school kind and helpful?	Never/ Rarely/ Sometimes (0), Most of the time/Always (1)
No Close friends	How many close friends do you have?	1 or more friends (0), No friends (1)
Alcohol use	During the past 30 days, on how many days did you have at least one drink containing alcohol?	0 days (0), 1 or more days (1)
Smoke cigarettes	During the past 30 days, on how many days did you smoke cigarettes?	No (0), 1 or more days (1)
Supportive parents or guardians	During the past 30 days, how often did your parents or guardians understand your problems and worries?	Never/ Rarely/ Sometimes (0), Most of the time/Always (1)

#### Individual level variables

The explanatory variables included in the analysis were selected based on previous research: they included age, sex, involvement in physical table 2

fighting, bullying victimization, serious injuries, socioeconomic status measured by hunger, parental support, helpful peers, substance use (cigarettes and/or alcohol), truancy, anxiety and loneliness. The questions used to measure the explanatory variables are defined in Table 1. These explanatory variables were also used as fixed effects variables for a multilevel model.

#### Country level variables

Seven country-level variables were identified for inclusion in the multilevel analysis. These included the Gini coefficient (GC), Gross Domestic Product (GDP) per capita, the pupil-to-teacher ratio in primary schools, the population density, the intentional homicide rate, laws regarding suicide and the night light index [11, 21, 26, 30, 32, 45, 48]. These variables were selected based on their importance as key socio-economic indicators as well as their potential to influence mental wel-being at population levels [4, 11, 41]. With the exception of a law criminalizing suicide (coded as No/Yes for each country), the other country-level variables were continuous. The pupil-to-teacher ratio represents a country’s investment in the education sector. This may imply that students receive adequate or inadequate support from their teachers based on the numbers of teachers to students per classroom or school.

**Table 2:** Included country data: Cross-national examination of adolescent suicidal behavior in 53 countries

Country	Year	Total	Total (12–16 year)	Ideation	Plan	Attempt
				*N* (W %, [95% CI])	*N* (W %, [95% CI9])	*N* (W %, [95% CI9])
Africa						
Benin	2016	2536	1174	162 (13.12, [10.05,16.96])	171 (13.97, [11.11,17.42])	153 (13.87, [11.03,17.31])
Ghana	2012	3632	1780	325 (18.90, [16.13,22.03])	384 (22.60, [18.34,27.53])	450 (26.69, [21.96,32.01])
Mauritania	2010	2063	1996	348 (17.42, [12.20,24.25])	300 (15.54, [11.82,20.17])	339 (17.06, [11.84,23.96])
Mozambique	2015	1918	1011	144 (15.51, [11.71,20.26])	161 (16.83, [12.55,22.20])	157 (16.27, [12.79,20.47])
Namibia	2013	4531	2655	521 (20.15, [17.91,22.59])	688 (25.53, [22.34,29.01])	698 (26.07, [22.02,30.56])
Seychelles	2015	2540	2470	507 (21.54, [19.60,23.62])	520 (21.95, [19.80,24.26])	477 (20.27, [17.65,23.16])
Tanzania	2014	3793	3093	372 (12.56, [10.95,14.37])	261 (8.64, [7.31,10.19])	316 (10.81, [8.82,13.19])
Subtotal		21,013	14,179	2379 (14.75, [13.59,15.99])	2 485 (13.77, [12.25,15.44])	2 590 (15.99, [14.10,18.08])
Americas						
Ant & Bar	2009	1266	1253	226 (17.21, [15.04,19.63])	233 (17.67, [15.48,20.08])	167 (12.22, [10.24,14.53])
Argentina	2012	28,368	28,055	4 972 (17.21, [15.73,18.79])	4 526 (15.94, [14.49,17.51])	4 463 (16.18, [14.95,17.48])
Bahamas	2013	1357	1343	235 (18.33, [15.79,21.18])	206 (15.45, [13.37,17.80])	185 (14.08, [11.80,16.71])
Belize	2011	2112	1972	289 (14.70, [12.97,16.61])	330 (17.25, [15.47,19.18])	264 (13.30, [11.39,15.48])
Bolivia	2012	3696	3497	640 (18.01, [16.11,20.08])	589 (16.84, [15.09,18.75])	730 (20.85, [18.91,22.92])
B. V. Is.	2009	1664	1589	234 (14.95, [14.95,14.95])	253 (15.99, [15.99,15.99])	202 (12.50, [12.50,12.50])
Costa Rica	2009	2679	2660	291 (10.87, [9.58,12.31])	200 (7.41, [6.53,8.41])	227 (8.46, [7.55,9.47])
Curaçao	2015	2765	1851	211 (11.70, [9.98,13.67])	173 (9.71, [8.03,11.71])	217 (11.94, [10.20,13.93])
El Salvador	2013	1915	1878	264 (13.84, [11.82,16.14])	209 (11.50, [9.48,13.90])	250 (13.18, [11.34,15.27])
Guyana	2010	2392	2361	530 (23.29, [21.07,25.66])	530 (23.12, [20.71,25.72])	
Honduras	2012	1779	1730	319 (19.44, [17.06,22.06])	315 (18.97, [16.81,21.33])	287 (17.21, [14.81,19.90])
Peru	2010	2 882	2853	576 (19.89, [17.92,22.02])	443 (15.27, [13.58,17.12])	492 (17.25, [15.80,18.80])
Suriname	2009	1698	1676	220 (13.53, [11.52,15.82])	192 (12.15, [10.17,14.45])	160 (9.92, [8.57,11.45])
Trinidad	2011	2811	2636	428 (16.51, [14.32,18.96])	447 (17.60, [15.33,20.12])	368 (13.56, [11.82,15.51])
Uruguay	2012	3524	3474	417 (12.33, [10.91,13.92])	379 (11.20, [9.65,12.97])	344 (10.15, [8.44,12.16])
Subtotal		60,908	58,828	9 852 (17.53, [16.72,18.37])	9 025 (15.29, [14.58,16.03])	8 356 (16.25, [15.55,16.97])
E. Mediterranean						
Afghanistan	2014	2579	1962	356 (19.66, [15.72,24.31])	302 (16.55, [13.27,20.44])	286 (14.77, [12.11,17.90])
Bahrain	2016	7141	6443	848 (14.89, [13.21,16.75])	835 (13.68, [12.15,15.38])	725 (12.69, [11.31,14.22])
Iraq	2012	2038	1988	331 (17.33, [15.02,19.90])	311 (16.57, [14.79,18.51])	323 (16.18, [14.10,18.49])
Kuwait	2015	3637	2689	410 (16.28, [13.81,19.11])	395 (16.22, [14.22,18.45])	406 (16.08, [12.86,19.92])
Lebanon	2011	2286	2249	328 (15.17, [13.20,17.37])	253 (11.48, [9.40,13.94])	305 (13.64, [11.77,15.75])
Morocco	2010	2924	2852	472 (16.49, [14.37,18.84])	412 (14.74, [12.75,16.97])	394 (13.98, [11.72,16.60])
Oman	2015	3468	2429	495 (21.00, [18.84,23.34])		
Pakistan	2009	5192	5170	375 (7.27, [6.28,8.41])	385 (7.56, [6.50,8.76])	
Palestine	2010	14,558	14,359	2 760 (20.42, [19.05,21.85])	2 369 (17.21, [16.39,18.06])	2810 (20.81, [19.80,21.86])
UAE	2010	2581	2561	387 (16.36, [14.21,18.77])	381 (15.88, [14.21,17.70])	356 (14.04, [12.11,16.22])
Subtotal		46,404	42,702	6762 (13.49, [12.48,14.57])	5643 (12.47, [11.56,13.44])	5586 (15.52, [14.36,16.75])

Country	Year	Total	Total (12–16 year)	Ideation	Plan	Attempt
				*N* (W %, [95% CI])	*N* (W %, [95% CI9])	*N* (W %, [95% CI9])
SE Asia						
Bangladesh	2014	2989	2949	124 (4.85, [3.66,6.42])	168 (7.45, [5.64,9.79])	197 (6.72, [5.25,8.57])
Indonesia	2015	11,142	9919	510 (4.89, [4.19,5.71])	558 (5.56, [4.86,6.36])	383 (3.87, [3.18,4.71])
Nepal	2015	6529	5727	700 (13.65, [11.31,16.38])	712 (13.68, [11.48,16.21])	576 (10.11, [7.84,12.93])
Thailand	2015	5894	4886	543 (12.15, [10.68,13.79])	608 (14.10, [11.93,16.59])	655 (13.59, [10.88,16.86])
Timor-Leste	2015	3704	2282	211 (9.33, [7.29,11.87])	229 (9.91, [7.80,12.51])	228 (10.08, [8.00,12.62])
Subtotal		30 258	25,763	2 088 (6.64, [5.99,7.37])	2 275 (7.89, [7.15,8.71])	1 879 (6.44, 5.67,7.31])
W. Pacific						
Brunei	2014	2599	2333	216 (9.24, [7.86,10.83])	149 (6.47, [5.32,7.86])	129 (5.57, [4.61,6.72])
Cook Is.	2015	701	532	74 (14.31, [11.95,17.05])	79 (14.55, [11.67,18.00])	65 (12.32, [9.14,16.40])
Fiji	2016	3705	2394	263 (11.35, [9.80,13.11])	305 (13.10, [11.16,15.32])	258 (10.88, [8.92,13.21])
Fr. Polynesia	2015	3216	2431	358 (14.22, [12.72,15.86])	411 (16.57, [15.14,18.11])	240 (9.96, [8.67,11.42])
Kiribati	2011	1582	1559	521 (34.49, [31.07,38.07])	514 (33.66, [30.52,36.94])	483 (31.43, [28.36,34.65])
Lao	2015	3683	2542	77 (3.06, [2.27,4.11])	116 (4.66, [3.79,5.71])	150 (5.92, [4.68,7.45])
Malaysia	2012	25,507	20,835	1484 (7.85, [7.19,8.57])	1189 (6.33, [5.86,6.83])	1420 (6.83, [6.12,7.62])
Mongolia	2013	5393	4442	995 (22.37, [20.63,24.22])	657 (14.73, [13.51,16.04])	437 (9.92, [8.98,10.95])
Nauru	2011	578	543	141 (27.81, [27.81,27.81])	118 (22.82, [22.82,22.82])	
Philippines	2015	8761	7793	792 (11.31, [9.91,12.89])	763 (10.67, [9.60,11.85])	1287 (16.54, [13.26,20.43])
Samoa	2011	2418	2 355	626 (33.67, [30.94,36.53])	781 (39.76, [36.13,43.52])	1354 (61.49, [55.21,67.40])
Solomon Is.	2011	1 421	1 332	371 (26.51, [22.01,31.56])	358 (26.32, [22.20,30.89])	441 (34.08, [26.62,42.42])
Tokelau	2014	140	101	25 (24.36, [19.12,30.51])	28 (27.26, [25.61,28.98])	27 (27.06, [20.87,34.30])
Tuvalu	2013	943	904	65 (7.46, [7.46,7.46])	95 (10.94, [10.94,10.94])	74 (8.31, [8.31,8.31])
Vanuatu	2011	1119	1024	181 (17.80, [14.01,22.35])	216 (20.72, [16.29,25.98])	253 (24.73, [20.10,30.04])
W&F	2015	1117	892	189 (22.09, [18.67,25.93])	242 (27.80, [24.87,30.93])	132 (15.09, [12.63,17.92])
Subtotal		62,883	52,012	6 378 (10.75, [9.67,11.92])	6 021 (9.84, [9.02,10.72])	6 529 (14.18, [11.68,17.11])
Total		221,466	193,484	27,459 (10.37, [9.92,10.83])	25,449 (10.27, [9.82,10.75])	24 940 (10.96, [10.28,11.68])

*N* unweighted number, *W %* weighted prevalence, *95% CI* confidence interval, *Ant & Bar* Antigua & Barbuda, *UAE* United Arab Emirates, *Cook Is.* Cook Islands, *Soloman Is.* Soloman Islands, *W&F* Wallis and Futuna, *B. V. Is.* British Virgin Islands

Population density has been linked with suicidal behavior at aggregate levels [21, 44]. Suicide and homicide rates appear to also be correlated suggestive of a wider socio-environmental stress component [17, 29]. Environmental factors may trigger pyschosocial stressors by way of insufficient social support structures, crime, and poverty. Crime also tends to heavily cluster in urban areas, particularly those on the lower end of the socioeconomic strata within societies characterized by high levels of socio-economic inequality [17, 29]. Lower suicide ideation rates have been found in countries where suicide attempts have been criminalized [32]. The night light index provides an indication of economic and human development within an area or region [2, 9, 14]. It is obtained using satellite images and takes population density into consideration [2]. The country estimates of GDP per capita, GC, homicide, population density, pupil-to-teacher ratios and the night light index were obtained from the United Nations and the World Bank [1, 2]. In the multilevel models, 33 countries that included all the country level variables were included in the analysis.

### Statistical analysis

A design-based analysis using a two-stage cluster weights was used in estimation the weighted prevalence of suicide by each country. The association between suicide related response variable and predictors (continuous and categorical) variables were assessed using survey *t* test and chi-square test respectively.

#### Multilevel mixed effects models

A two-level multivariable multilevel mixed effects logistic regression model was fitted assess the risk factors for suicide related responses. The mixed effects model was fitted with fixed individual and country level covariates and random country level intercepts. The model was fitted based on Hox’s 2010 bottom-up approach [19].

The first step involved the intercept only model that predicted having considered suicide, made a plan or attempted suicide separately. Thereafter, the fixed level of individual and country explanatory factors were added to the model with those found to be significant being retained. However, the country level variables were retained as fixed effects regardless of statistical significance. The effects model allows for heterogeneity to be taken into consideration, at the different levels of the data structure [13].

The variables with significant group level variations were also tested for cross level interactions at each level. Interactions between the country indicators and the significant individual variables were retained in the final model if significant.

A Gauss–Hermite adaptive quadrature approach was employed to estimate the mixed effects model parameters and the fit was assessed using Bayesian Information criterion. The level of statistical significance was at $p<$0.05 with the confidence interval reported at 95%. Stata version 17 (StataCorp, TX, USA) was used for the analysis.

## Results

**Table 3:** Descriptive statistics for the fixed effect variables

Variable	Number (weighted data)	Percent (weighted)
Sex (male)	99,724	51.89
Hunger	12,863	6.71
Attacked	73,529	38.37
Physical fight	60,131	31.29
Injured	67,986	40.41
Bullying victimization	58,406	32.07
Lonely	19,821	10.39
Anxiety	14,580	7.60
No close friends	11,156	5.87
Use of Cigarettes /alcohol	30,698	16.03
Truancy	52,039	27.56
Helpful peers	77,454	41.14
Supportive parents or guardians	71,403	37.92

**Table 4:** Estimates from Mixed effects logistic regression model: Cross-national examination of adolescent suicidal ideation behavior in 33 countries

Variable	Adjusted odds ratio	95% Confidence interval	*p* value
Age	1.06	1.04, 1.08	< 0.001
Sex (ref: Male)	1.90	1.82, 1.99	< 0.001
Went hungry	1.13	1.04, 1.22	0.004
Attacked	1.40	1.33, 1.46	< 0.001
Physical fighting	1.22	1.16, 1.28	< 0.001
Injured	1.31	1.25, 1.37	<0.001
Bullying victimisation	1.58	1.51, 1.66	< 0.001
Loneliness	2.77	2.63, 2.93	< 0.001
Anxiety	2.27	2.14, 2.42	< 0.001
No close friends	1.58	1.46, 1.71	< 0.001
Truancy	1.25	1.19, 1.30	< 0.001
Helpful peers	0.87	0.83, 0.90	< 0.001
Supportive parents or guardians	0.65	0.62, 0.68	< 0.001
Alcohol/smoke cigarettes	1.97	1.87, 2.06	< 0.001
Gini coefficient	1.00	0.98, 1,03	0.888
GDP per capita	1.04	0.98, 1.11	0.234
Pupil to teacher ratio	1.00	0.97, 1.02	0.839
Homicide	1.01	1.00, 1.02	0.060
Population density	0.95	0.85, 1.05	0.277
Availability of suicide law	1.04	0.68, 1.58	0.865
Night light index	5.13	0.42, 62.70	0.200
Random effects	Variance		
Intercept (country level)	0.19	0.12, 0.32	<0.001

A total of 193,464 adolescents aged 12–16 years were included in the analysis (Table 2). Approximately 19,551 (10.37%) participants considered attempting suicide, 19,166 (10.27%) made a suicide plan and 19,819 (10.96%) attempted suicide. By country, suicide ideation prevalence ranged from 3.06% in Lao to 33.67% in Samoa, with both countries being located within the Western Pacific region [10, 46]. Similarly, the prevalence of making a suicide plan ranged from 4.66% in Lao to 39.76% in Samoa. While the attempted suicide prevalence ranged from 3.87% in Indonesia to 61.49% in Samoa. By gender, approximately 55.06% of the females had considered suicide, while 52.79% of females had made a plan, and 51.51% of females attempted suicide. Roughly 40% of the adolescents reported being seriously injured at least once, 31.29% reported having engaged in physical fights, while 32.07% were bullied (Table 3).

In the multilevel model, among adolescents who had considered suicide (Table 4), the highest odds were among teens experiencing loneliness (OR 2.77, 95% CI 2.63, 2.93; $p<$ 0.001), anxiety (OR 2.27, 95% CI 2.14, 2.42; $p<$ 0.001) and the use of alcohol and/or smoking cigarettes (OR 1.97, 95% CI 1.87, 2.06; $p<$ 0.001). Students with helpful peers at school and supportive parents or guardians were less likely to report suicide ideation at (OR 0.87, 95% CI 0.83, 0.90; $p<$ 0.001) and (OR 0.65, 95% CI 0.62, 0.68; $p<$ 0.001) respectively. No significant interactions were found between the country and individual level variables.

Among adolescents who made a suicide plan (Table 5), the strongest individual risk factors were among teens had felt lonely (OR 2.32, 95% CI OR 2.19, 2.46; $p<$ 0.001), anxious (OR 2.05, 95% CI 1.92, 2.18; $p<$ 0.001), had no close friends (OR 2.03, 95% CI 1.88, 2.19; $p<$ 0.001) and used alcohol and/or smoked cigarettes (OR 1.85, 95% CI 1.76, 1.94; $p<$ 0.001). Students who had supportive and understanding parents or guardians were also less likely to make suicide plans (OR 0.72, 95% CI 0.68, 0.75; $p<$ 0.001) and those with helpful peers at school (OR 0.93, 95% CI 0.89, 0.97; *p* = 0.001). For every increase in the night light index, there were higher odds of making a suicide plan (OR 15.44, 95% CI 1.94, 122.97; *p* = 0.010). No significant interactions were found between the country and individual level variables.

Congruently, among adolescents who attempted suicide (Table 6), the strongest individual risk factors included loneliness (OR 2.10, 95% CI 1.98, 2.23; $p<$ 0.001), feeling anxious (OR 2.06, 95% CI 1.93, 2.20; $p<$ 0.001), a lack of close friends (OR 2.02, 95% CI 1.87, 2.19; $p<$ 0.001) and the use of alcohol and/or smoking cigarettes (OR 1.96, 95% CI 1.87, 2.07; $p<$ 0.001). The teenagers were less likely to attempt suicide if they had supportive parents or guardians (OR 0.77, 95% CI 0.73, 0.81; $p<$ 0.001) and helpful peers at school (OR 0.90, 95% CI 0.86, 0.94; $p<$ 0.001). The night development index was associated with attempting suicide, however with a very wide confidence interval (OR 36.67,95% CI 2.69, 500.36; *p* = 0.007). No significant interactions were found between the country and individual level variables.

## Discussion

**Table 5:** Estimates from Mixed effects logistic regression model: Cross-national examination of adolescent’s making a suicidal plan behavior in 33 countries

Variable	Adjusted OR	95% CI	*p* value
Age (continuous)	1.04	1.02, 1.06	< 0.001
Sex (ref: Male)	1.64	1.57, 1.72	< 0.001
Went hungry	1.09	1.01, 1.19	0.030
Attacked	1.36	1.29, 1.43	< 0.001
Physical fighting	1.24	1.18, 1.30	< 0.001
Bullying victimisation	1.45	1.39, 1.52	< 0.001
Loneliness	2.32	2.19, 2.46	< 0.001
Anxiety	2.05	1.92, 2.18	< 0.001
No close friends	2.03	1.88, 2.19	< 0.001
Truancy	1.27	1.21, 1.33	< 0.001
Helpful peers	0.93	0.89, 0.97	0.001
Supportive parents or guardians	0.72	0.68, 0.75	< 0.001
Alcohol/smoke cigarettes	1.85	1.76, 1.94	< 0.001
Gini coefficient	1.01	0.99, 1.03	0.463
GDP per capita	1.04	0.99, 1.10	0.155
Pupil to teacher ratio	0.99	0.98, 1.01	0.584
Homicide	1.01	1.00, 1.02	0.077
Population density	0.99	0.91, 1.07	0.761
Availability of suicide law	0.92	0.65, 1.30	0.620
Night light index	15.44	1.94, 122.97	0.010
Random effects	Variance		
Intercept (country level)	0.13	0.08, 0.22	< 0.001

Approximately 10% of the adolescents in the present study reported having had suicidal thoughts, with (10%) having made a suicide plan, while 11% had attempted suicide. Except for the night light index, the country level variables were not associated with suicidal behavior.

The prevalence of suicide ideation was similar to that reported in a multi-national study by Koyanagi et al. [22] but also lower than reported by Page et al and Uddin et al, who had rates ranging from 15 to 17% [40, 46]. The prevalence rate for adolescents who made a suicide plan was lower than the 17% rate reported by Uddin et al. [46], but higher than reported by McKinnon et al. at 8.3% [27]. The attempted suicide prevalence was similar to that reported by Koyanagi et al. [22] but lower than reported by both Liu et al. and Uddin et al. at 17% [25, 46].

By region, South East Asia had the lowest prevalence for the three categories of suicidal behavior as reported by Liu et al and Uddin et al. [25, 46]. Our study also found the Americas had the highest rates which is in contrast to what was reported by Page et al., Uddin et al and McKinnon et al. who found the African region had the highest rates for ideation [27, 40, 46] and planning [40, 46]; while the Western Pacific had the highest rates for attempted suicide [25, 46]. The difference in the highest prevalence rates could be attributed to the timing (survey year) of the GSHS, and subsequently the number of countries that were selected for inclusion by the WHO regions within the research studies. The total population included in a study ultimately influences the prevalence rates that are estimated given the population numerators and denominators used. For instance, from the African region Page et al, Uddin et al and McKinnon et al reported high suicide ideation rates in Benin (2009—22%), Kenya (2003—28%) and Zambia (2003—31%), and suicide planning rates of 30%, 30% and 41% in 2003, respectively [27, 40, 46]. We included data from 2009–2016 and thus excluding Kenya and Zambia, but used the Benin 2016 survey data which had lower prevalence rates at 13% than in 2009 [27, 40]. This may have contributed to the lower suicide ideation rates observed in the African region. Regarding the studies that reported the highest attempted suicide as being from the Western Pacific nations, Liu et al. included six countries [25], Uddin et al. used 10 countries [46] in comparison to the current study that included 16 countries. The variations in the number of countries selected in the studies may have influenced the prevalence rates for attempted suicide. However, the prevalence rates by country for the same years in this study were similar across the multi-national studies reported by Uddin et al and Liu et al. [25, 46]. Overall, there were notable differences in prevalence by gender, however, there was no gender difference in suicide ideation, making a suicide plan and attempting suicide from the African and East Mediterranean regions as reported by Liu et al. and Uddin et al. [25, 46].

The country level covariates (the GC, GDP per capita, the pupil-to-teacher ratio in primary schools, the population density, the intentional homicide rate, the law on suicide and the night light index) were not significant in the random effect models. The income level findings are consistent with studies conducted by Carpena et al and Tan et al in Brazil and China that found no association between suicidal thoughts and the GDP nor the GC [11, 45]. Conversely, studies by Bando et al and Santurtn et al from Brazil and Spain found an inverse association between suicide mortality and GDP [4, 41], but also a positive association [4]. While it is anticipated that income inequality may contribute to higher suicidal behavior among poorer communities, social cohesion through supportive families in these areas may help mitigate some factors associated with suicidal tendencies [45].

There are conflicting results regarding population density and suicidal behavior. Werneck et al and Stark et al found an increased likelihood of suicide ideation and mortality being associated with a higher population density in Brazil and Scotland [44, 48]. The inverse was reported by Knipe et al in Sri Lanka, for example, in rural areas with a low population density [21]; while no association was found O’Reilly et al in Northern Ireland as well [36]. Urban areas represent employment opportunities but could also potentially expose inhabitants to mental health problems due to pressures related to increased competition for jobs, affordable housing and health care [48]. Furthermore, areas that are rural and with a lower population density may have higher unemployment rates which could exacerbate distress among individuals [21, 45].

Homicide and suicidal behavior are significantly correlated in Europe [7, 17, 26], but not in the Americas and Asia [7, 17] based on global research studies. Within the regional studies, the direction of the associations between suicidal behavior and homicide depended on geographical locations with positive associations mostly observed in Europe and no or a negative association in the Americas [7, 17]. It is expected that larger cities or poorer neighborhoods are more likely to have higher homicide rates due to various factors which could be influenced by income levels, inequality and access to basic services.

Additionally, it is envisaged that a lower pupil-to-teacher ratio improves learning outcomes [45]. A study conducted by Tan et al in China found a negative association for the pupil-to-teacher ratio [45] meaning a higher ratio was protective against suicide ideation. However, it was suggested that the pupil-to-teacher ratios could have influenced the academic performance of the students instead [45]. Class sizes tend to influence learning outcomes among students, and students may freely engage with teachers in matters related to academics, socio-cultural or health, including mental health depending on the availability of teachers.

The night light development index was associated with suicidal behavior. This is consistent with a South Korean study which found the exposure to artificial night light was associated with an increased risk of suicidal behavior and depressive symptoms [30]. There are concerns that night light could contribute to psychological issues such as insomnia, reduced production of melatonin, mood disorders and metabolic changes, thus affecting mental health [30]. The night light index is a proxy for economic development and human development in an area [9]. Considering that other economic indicators like GDP and the gini coefficient were not significant, it is surprising that the night light index was associated with suicidal behavior. The timing of the data collection for these national indicators and the surveys may not have overlapped, hence the discrepancies.

Countries that criminalize suicide attempt do not necessarily have lower rates of suicide compared to those where it is decriminalized [32]. We found no association with the presence of a criminal law on suicidal behavior. Lester et al found an increase in the official suicide statistics after decriminalization in seven countries; however, no significant change was found in Ireland, Canada and New Zealand [23, 38]. Attempting suicide is criminalized in about 45 countries [32, 38]. It is therefore possible that suicidal behavior may be under reported due to stigma or fear of criminal prosecution [32]. However, evidence suggests that involvement in extensive social support networks, may serve as coping mechanisms. Individuals tend to socially receive support thus potentially explaining the small or comparable gender differences observed [39]. Additionally, females are more likely to take advantage of social support by seeking help, which could alleviate some stress they may encounter [31]. Moreover, the small gender differences in the East Mediterranean region may be also be affected by social support networks as well as religion [12]. It is important for mental health support to be offered to suicidal individuals rather than punishments.

This multilevel study sought to examine if any of the seven identified contextual country level variables were associated with suicidal behavior in addition to the individual risk factors and prevalence rates. The previous studies by Page et al, Uddin et al and Koyanagi, however, focused mainly on prevalence, without delving too deeply into socio-demographic factors predisposing survey participants to suicidal behavior [22, 40, 46]. On the other hand, the studies by McKinnon et al, Campisi et al and Liu et al estimated both the prevalence and risk factors for suicide behavior [10, 25, 27]. Liu et al conducted a multilevel analysis and used GDP as a variable of interest in addition to the fixed effect variables [25]. We found no significant interactions between the country level factors and the individual level factors. O’Reilly et al similarly found no interactions between individual level factors (age and sex) and area level factors like population density [36]. Although O’Reilly et al was a 5-year follow up study and attributed it to insufficient power, our study had sufficient power. Stark et al found interactions between time period and deprivation [44]. Our study was derived from cross-sectional studies thus the interactions with time were not tested.

### Strengths and limitations

**Table 6:** Estimates from Mixed effects logistic regression model: Cross national examination of adolescent’s suicidal attempt in 33 countries

Variable	Adjusted OR	95% CI	*p* value
Age (continuous)	1.01	0.99, 1.03	0.321
Sex (ref: Male)	1.66	1.59, 1.74	< 0.001
Went hungry	1.24	1.15, 1.35	< 0.001
Attacked	1.42	1.36, 1.50	< 0.001
Physical fighting	1.33	1.27, 1.40	< 0.001
Injured	1.50	1.44, 1.58	< 0.001
Bullying victimisation	1.71	1.63, 1.79	< 0.001
Loneliness	2.10	1.98, 2.23	< 0.001
Anxiety	2.06	1.93, 2.20	< 0.001
No close friends	2.02	1.87, 2.19	< 0.001
Truancy	1.40	1.34, 1.47	< 0.001
Helpful peers	0.90	0.86, 0.94	< 0.001
Supportive parents or guardians	0.77	0.73, 0.81	< 0.001
Alcohol/smoked cigarettes	1.96	1.87, 2.07	< 0.001
Gini coefficient	1.02	0.99, 1,04	0.141
GDP per capita	1.04	0.97, 1.11	0.274
Pupil to teacher ratio	0.99	0.97, 1.01	0.434
Homicide	1.01	1.00, 1.02	0.191
Population density	1.00	0.90, 1.11	0.976
Availability of suicide law	0.95	0.61, 1.47	0.809
Night light index	36.67	2.69, 500.36	0.007
Random effects	Variance		
Intercept (country level)	0.21	0.13, 0.34	< 0.001

The study used data from a standardized survey that was nationally representative and validated internationally. Additionally, it represented different regions from LMICs from which data tends to be limited. Unlike previous multinational studies, this study examined the country and contextual differences within multilevel models to identify associations. Thus, although there are contradicting results regarding contextual factors, this study remains silent on the associations between suicidal behavior. Among the limitations, adolescents who did not attend school on the day the survey was administered were not represented in the study. Additionally, assuming the out of school adolescents have different challenges, they may not have been adequately represented. The factors in countries where gender differences exist in the school attendances may also not have been well captured. Furthermore, the study used self-reported questionnaires which may be prone to recall and social desirability bias and would require interpretation with caution. Moreover, owing to stigma surrounding mental health and suicide in various countries or communities, it is possible that the respondents may have modified their responses to suit their situations.

## Conclusion

The prevalence of suicidal behavior varied by country and geographical region. Several individual level variables were significantly associated with suicidal behavior. However, the country-level contextual variables were found to not be statistically significant. These findings fall against the backdrop of continuous efforts aimed at assessing and monitoring suicidal behavior globally. It is anticipated that they may have the potential to support decision-making with regard to what factors at individual and country levels warrant consideration in follow-up studies. The individual level findings suggest that individualized mental health and social support represent important components in addressing suicidal behavior, irrespective of socio-economic context. The non-significant country level findings were not entirely surprising given the mixed results from prior studies. Additional information has been highlighted with regard to the country level factors associated with suicidal behavior across adolescent populations.
